# Sleep in Autism Across the Lifespan: A Protocol for a Cross-Sectional Survey with Nationwide Dissemination in Spain

**DOI:** 10.3390/healthcare14101398

**Published:** 2026-05-20

**Authors:** María Luisa Sánchez de Ocaña-Moreno, Ana María García-Muñoz, Isabel María Timón, Guillermo Benito Ruiz, Marta Plaza Sanz, Ruth Vidriales Fernández, Elena Martínez-Cayuelas, Laura Gisbert-Gustemps, Jorge Lugo-Marín, Gonzalo Pin-Arboledas, Isabel Mengual-Luna, Juana Mulero-Cánovas, Pilar Zafrilla, Begoña Cerdá, Beatriz Rodríguez-Morilla, Pura Ballester-Navarro

**Affiliations:** 1 Health Sciences PhD Program, Universidad Católica de Murcia UCAM, Campus de los Jerónimos nº135, 30107 Murcia, Spain; 2 Faculty of Pharmacy and Nutrition, Universidad Católica de Murcia UCAM, 30107 Murcia, Spain; 3 Department of Sociosanitary Sciences, University of Murcia, 30120 Murcia, Spain; 4 Spanish Autism Confederation, 28007 Madrid, Spain; 5 Department of Pediatrics, Health Research Institute–Fundación Jiménez Díaz, Autonomous University of Madrid, 28040 Madrid, Spain; 6 Department of Psychiatry, Vall d’Hebron University Hospital (HUVH), 08035 Barcelona, Spain; 7 VHIR: Psychiatry, Mental Health and Addictions Group, Vall d’Hebron Research Institute (VHIR), Instituto de Investigación Sanitaria Acreditado Instituto de Investigación—Hospital Universitario Vall d’Hebron, 08035 Barcelona, Spain; 8 Biomedical Research Networking Centre in Mental Health (CIBERSAM), 28029 Madrid, Spain; 9 Sleep and Chronobiology Committee of the Spanish Association of Pediatrics (AEP), 28008 Madrid, Spain; 10 Faculty of Education, Universidad Católica de Murcia UCAM, 30107 Murcia, Spain; 11 Chronobiology Laboratory, IMIB-Arrixaca, University of Murcia, 30120 Murcia, Spain; 12 Kronohealth SL, 30100 Murcia, Spain

**Keywords:** autism spectrum disorder, intellectual disability, sleep disturbances, age

## Abstract

**Highlights:**

**What are the main findings?**

- This study presents a cross-sectional protocol with nationwide dissemination to assess sleep patterns in individuals with autism across childhood, adolescence, and adulthood in Spain.
- Sleep is evaluated using validated, age-appropriate questionnaires adapted for intellectual ability, together with internalizing symptoms and other clinical comorbidities that may affect sleep.

**What are the implications of the main findings?**

- The protocol enables the identification of vulnerable sleep profiles across different life stages in the autistic population.
- The resulting findings may inform targeted clinical screening and tailored interventions to improve sleep and overall well-being in individuals with autism.

**Abstract:**

**Background:** Autism spectrum disorder (ASD) is consistently associated with a high prevalence of sleep disturbances across the lifespan, with reported rates ranging from 60% to 86% depending on age and clinical characteristics. Although this issue has been widely described in the international literature, Spain currently lacks large-scale data to estimate the prevalence of sleep disturbances or to examine their relationship with factors such as age, intellectual disability, and co-occurring conditions. This study aims to estimate the prevalence and severity of sleep disturbances in individuals with autism spectrum disorder in Spain and to examine their associations with developmental stage, intellectual disability, affective symptoms, and contextual factors. **Methods:** This is a cross-sectional observational survey with nationwide dissemination approved by the Ethics Committee of the Universidad Católica San Antonio de Murcia. Data will be collected through an online survey (SurveyMonkey) including validated instruments: the Children’s Sleep Habits Questionnaire–Autism (CSHQ-Autism) and the Sleep Disturbance Scale for Children (SDSC) for pediatric participants; the Pittsburgh Sleep Quality Index (PSQI) for adolescents and adults without intellectual disability; and the Diagnostic Assessment for the Severely Handicapped–II (DASH-II) for adults with intellectual disability. Anxiety and depressive symptoms will be assessed using the Child Behavior Checklist (CBCL) in children and adolescents and the Hospital Anxiety and Depression Scale (HADS) and DASH-II. Statistical analyses will be conducted using SPSS v22 by applying parametric or non-parametric tests according to data distribution. **Conclusions:** This study represents one of the first survey protocols with nationwide dissemination designed to assess sleep disturbances in individuals with ASD in Spain. The resulting findings are expected to help identify vulnerability profiles, inform public health strategies, and support the development of multidisciplinary interventions aimed at improving sleep and, consequently, the quality of life of individuals with autism and their families.

## 1. Introduction

Autism spectrum disorder (ASD) is a neurodevelopmental condition characterized by persistent deficits in social communication and interaction, alongside repetitive and ritualized patterns of behavior and restricted interests or activities [1]. In accordance with the preferences expressed by individuals with this diagnosis, this manuscript adopts identity-respecting language and uses the term “person with autism” [2]. From a contemporary perspective, autism is increasingly conceptualized within a multidimensional framework that integrates biological, psychological, and environmental factors, which may interact to shape functional outcomes across the lifespan.

The prevalence of ASD has recently been updated to approximately 1 in 36 live births [3], with estimated adult prevalence rates ranging from 1.97% to 2.42% when accounting for undiagnosed cases [4]. Individuals with autism frequently present with coexisting medical and psychiatric conditions, among which sleep problems represent a lifelong challenge [5]. The scientific community increasingly encourages the analysis of sleep disturbances in autism from medical [6], psychosocial, and environmental perspectives [7]. However, despite their high frequency, large-scale prevalence data on sleep problems among people with autism in Spain remain scarce.

International research consistently reports a high prevalence of sleep disturbances across all developmental stages in individuals with autism. During childhood, prevalence rates may reach up to 80% [8], confirming their persistence throughout adolescence (40–80%) [9,10,11] and into adulthood (45% approximately) [12,13,14,15], regardless of the presence or absence of co-occurring intellectual disability. The most frequently reported sleep problems are insomnia symptoms, affecting between 60% and 86% of individuals depending on the age group [16]. These symptoms primarily manifest as difficulties initiating sleep and a high number of nocturnal awakenings [7,17,18,19]. Circadian rhythm disruptions are also commonly reported, including delayed sleep phase across all age groups [20] and, conversely, advanced sleep phase in institutionalized adults with autism [21]. This misalignment between the endogenous circadian system and environmental cycles [22] may be associated with core ASD characteristics that affect the ability to interpret social cues, thereby impacting participation in daily life activities [23].

Despite the robustness of international evidence summarized above, there is a lack of large-scale studies using nationwide dissemination strategies to examine sleep disturbances among individuals with autism in Spain. Existing research in this context is limited in scope and often based on small or clinically selected samples, which restricts the generalizability of findings. This gap limits the ability to understand how sleep disturbances may vary according to age, intellectual disability status, emotional symptoms, and contextual factors within the Spanish population. To estimate the prevalence of sleep disturbances in a geographically diverse sample of individuals with autism in Spain, validated questionnaires will be used in the present study. In children with ASD, the Children’s Sleep Habits Questionnaire (CSHQ) is widely applied [24]. In the present study, the autism-adapted version (CSHQ-Autism) will be used [25], alongside the Sleep Disturbance Scale for Children (SDSC) to provide a broader and more longitudinal perspective [26]. Adolescents and adults without intellectual disability will complete the Pittsburgh Sleep Quality Index (PSQI) [27], while adults with autism and co-occurring intellectual disability will be assessed through caregiver-reported responses using the Diagnostic Assessment for the Severely Handicapped–II (DASH-II) [28].

The prevalence of sleep problems among individuals with autism is consistently high across all stages of life. Evidence suggests that factors such as psychological comorbidities and living conditions significantly influence sleep in this population. The research is guided by the following hypotheses: (a) sleep disturbances will show a high prevalence and clinically significant severity across all age groups in individuals with autism spectrum disorder in Spain; (b) the prevalence and severity of sleep disturbances will differ according to developmental stage and intellectual disability status, with more severe and complex sleep problems expected in individuals with co-occurring intellectual disability; (c) higher levels of anxiety and depressive symptoms will be positively associated with greater severity of sleep disturbances across all age groups; (d) contextual and clinical variables (e.g., treatment status, living conditions) will be significantly associated with sleep outcomes, independently of age and sex; (e) in multivariable models, affective symptoms and contextual factors will remain significant predictors of sleep disturbances after adjusting for demographic and clinical covariates; (f) distinct vulnerability profiles will be identifiable, characterized by the co-occurrence of severe sleep disturbances with specific combinations of demographic (e.g., age), clinical (e.g., intellectual disability), and affective factors; and (g) estimates of sleep disturbances may differ according to respondent type (self-report vs. proxy-report), although the overall pattern of associations is expected to remain consistent in stratified analyses. Therefore, this study aims to estimate the prevalence and severity of sleep disturbances among individuals with autism in Spain and to examine their variability according to developmental stage, intellectual disability status, affective symptoms, and contextual or clinical factors. By addressing these questions, the study seeks to generate epidemiological evidence from a geographically diverse Spanish sample and to provide a foundation for future longitudinal and interventional research.

## 2. Materials and Methods

### 2.1. Study Design

This study is designed as an observational, cross-sectional survey with nationwide dissemination aimed at examining sleep characteristics in individuals with ASD living in Spain. The cross-sectional design allows the simultaneous collection of data from participants at a specific point in time, enabling the estimation of the prevalence and distribution of sleep disturbances within the study population.

The project is conducted within the framework of the PhD programme in Health Sciences at the Universidad Católica San Antonio de Murcia. The questionnaire was developed in collaboration with experts from the Fundación Jiménez Díaz, Vall d’Hebron University Hospital, the Spanish Autism Confederation, and the pharmaceutical laboratory Exeltis.

The study protocol was approved by the Ethics Committee of the Universidad Católica San Antonio de Murcia in February 2023 (approval code: CE22303). All procedures will be conducted in accordance with the ethical principles established in the Declaration of Helsinki and with current regulations regarding research involving human participants. Ethical approval ensured that the study met the necessary standards related to participant protection, confidentiality, and responsible data management.

### 2.2. Participants and Eligibility Criteria

The target population of this study comprises individuals with ASD living in Spain across different stages of life, including children, adolescents, and adults, with or without co-occurring intellectual disability. ASD diagnosis will be ascertained through several specific and mandatory questions included in the survey. Respondents will be required to select the diagnosis received from a predefined list and to provide additional clinical information related to the diagnosis. This information will be reviewed by an expert clinician with experience in ASD to assess its consistency and plausibility. Responses with non-plausible or internally inconsistent diagnostic information will be excluded from the database. However, as this is a survey-based protocol, ASD diagnosis will not be independently verified through clinical records. Participants will be recruited through the Spanish Autism Confederation, which is closely connected to autism-related associations across the national territory, as well as through clinical ASD networks and support organizations across the country. These organizations will disseminate the survey link directly to eligible participants (individuals with a prior ASD diagnosis or their caregivers) via email and WhatsApp distribution lists. This approach allows the study to reach a geographically diverse sample; however, we acknowledge that it relies on convenience sampling and may introduce selection bias.

Eligible participants will be those residing in Spain with a prior diagnosis of ASD and sufficient information available to complete the survey pathway corresponding to their age group and cognitive profile. Participants will not be excluded on the basis of the presence or absence of sleep problems, psychiatric comorbidities, medical conditions, or intellectual disability, as the aim of the study is to characterize sleep disturbances and their associated clinical and contextual correlates in a broad and heterogeneous ASD population. This inclusive approach is intended to improve the external validity of the study and to better reflect the diversity of real-world clinical presentations.

Exclusion criteria will include failure to provide informed consent, residence outside Spain, absence of a previous ASD diagnosis, and questionnaires with insufficient information in key sections required for analysis. In addition, duplicate or internally inconsistent responses identified during the data-cleaning process will be excluded from the final analytical sample.

### 2.3. Sample Size Calculation

The sample size was estimated for a finite population using parameters appropriate for prevalence studies, as the primary objective is to estimate the prevalence of sleep problems in individuals with autism spectrum disorder (ASD) and to examine differences across clinically relevant subgroups. Because the total source population is known, the finite population correction was applied. The calculation incorporated both the reported prevalence of ASD in the reference population [3] and previously published estimates of sleep problems in this group (45–79%) [8,9,10,11,12,13,14,15], selecting a conservative expected prevalence (p) to maximize the required sample size and thus ensure adequate precision.

The following formula was applied: *n = [N × Z^2^ × p × (1 − p)]/[d^2^ × (N − 1) + Z^2^ × p × (1 − p)]*, where *N* is the total population size, *Z* = 1.96 for a 95% confidence level, *p* is the expected prevalence, *q = 1 − p*, and *d* = 0.05 is the desired precision. This yielded a minimum sample size of 383 participants for the overall prevalence estimate. To maximize variance and ensure a conservative estimate, an expected prevalence of *p* = 0.50 was used in the final calculation. The calculation assumed a two-sided significance level of *α* = 0.05, corresponding to *p* < 0.05.

Importantly, because the analytical plan includes subgroup analyses by age group and intellectual disability status, the sample size estimation was further informed by expected subgroup-specific prevalences of sleep problems. To mitigate the loss of statistical power in these stratified analyses, recruitment will intentionally exceed the minimum required sample size to ensure sufficient representation within each subgroup. A minimum of 90 participants was established for each age group, with an approximate ratio of 1 participant with intellectual disability to every 2 participants without intellectual disability, reflecting the distribution observed in the target population. Additionally, age groups will be defined to avoid excessively small strata, and where necessary, categories may be combined to preserve analytical robustness.

Given that data will be collected through an online survey and that some responses may be excluded due to incompleteness, duplication, or inconsistency, oversampling will be employed. This approach is expected to preserve both the overall precision of prevalence estimates and adequate statistical power for subgroup comparisons.

### 2.4. Variables Measured in the Online Survey

Data will be collected through an online survey administered via the SurveyMonkey^®^ platform, consisting of a total of 139 items. However, due to an adaptive branching structure based on participants’ age and the presence or absence of intellectual disability, each participant or their legal guardian will complete approximately 50–60 questions. This branching design ensures that participants will only be presented with questions relevant to their specific demographic and clinical characteristics. As a result, the questionnaire remains manageable in length while still collecting comprehensive information on sleep patterns and associated variables.

The survey includes variables related to sociodemographic characteristics, clinical profile, sleep habits and disturbances, affective symptoms, and other contextual factors considered relevant to the study aims. This structure allows the assessment of sleep-related features across different developmental stages and levels of functioning while minimizing respondent burden.

Completion of the online survey requires approximately 20 min per participant. For children, adolescents, and adults with intellectual disability, questionnaires will be completed by parents or legal guardians (proxy report), whereas cognitively able adult participants will complete the survey themselves (self-report). This mixed-informant approach is necessary to avoid systematic exclusion of individuals with intellectual disability and to improve the coverage of the target population. Given the known differences between self- and proxy-reported outcomes, the type of respondent (self vs. caregiver) will be explicitly recorded and incorporated into the analytical strategy. Analyses will be restricted to comparable measures across informant types, and no direct comparisons will be made between self- and proxy-reported responses unless measurement equivalence can be reasonably assumed (e.g., diagnosis). When subgroup analyses are conducted (e.g., by age or intellectual disability status), they will be aligned with the type of respondent to maintain internal consistency. To assess the potential impact of mixed informants, sensitivity analyses will be performed by stratifying results according to respondent type. This approach prioritizes inclusivity while preserving interpretability and analytical validity.

#### 2.4.1. Common Questionnaire

The common questionnaire constitutes the core component of the survey and serves to establish subsequent filtering pathways. It consists of 22 items collecting general demographic and clinical information, including age, sex, educational status, diagnostic characteristics of individuals with ASD, and associated treatments. This information provides an initial comprehensive profile of the participating population, forming the basis for subsequent analyses of sleep-related outcomes and comorbidities.

#### 2.4.2. Age-Adapted Questionnaires

Based on the initial common section and the filtering criteria established therein, this part of the survey includes age- and cognitive ability–specific instruments for the assessment of sleep and associated disorders.

***Questionnaire for children aged 0–12 years***

Sleep in children aged 0–12 years, with or without co-occurring intellectual disability, will be assessed using the CSHQ, which consists of 32 items and is based on three response options, each associated with a specific score. The possible responses for each item are: usually (occurring 5–7 times per week; 3 points), sometimes (2–4 times per week; 2 points), and rarely (0–1 times per week; 1 point) [25,29,30].

More recently, a modified version of this instrument, the CSHQ–Autism, has been developed, consisting of 23 items with a particular focus on behavioral insomnia, excluding items related to sleep-disordered breathing [25,29,30].

Anxiety and depressive symptoms will be assessed using a shortened 19-item version of the Child Behavior Checklist (CBCL) [31]. CBCL items will be treated as ordinal categorical variables using a three-point response scale: 0 = Not true (the statement does not describe the child), 1 = Somewhat or sometimes true, and 2 = Very true or often true.

***Questionnaire for adolescents aged more than 12 years up to 18 years without co-occurring intellectual disability***

Sleep will be assessed using the self-reported PSQI, which consists of 19 items grouped into seven components, each scored from 0 to 3. The scores of the seven components are summed to obtain a global score ranging from 0 to 21, with higher scores indicating poorer sleep quality. A score of 0 reflects the absence of sleep difficulties, whereas a score of 21 indicates severe sleep problems across all assessed domains [32].

***Questionnaire for adolescents aged more than 12 years up to 18 years with co-occurring intellectual disability***

Sleep will be assessed using the SDSC, which consists of 27 items designed to detect sleep disorders by evaluating sleep-related behaviours over the previous six months. This instrument is characterised by its simplicity and ease of administration, as well as by its straightforward scoring system for identifying potential sleep disturbances [26].

***Questionnaire for adults ≥ 18 years without co-occurring intellectual disability***

Sleep quality will be assessed using the PSQI, with the global sleep quality score calculated from seven subscales, as previously described [33]. Each subscale evaluates a different aspect of sleep quality, and the combined score provides an overall indicator of sleep disturbances.

Anxiety and depressive symptoms will be assessed using the Hospital Anxiety and Depression Scale (HADS), a brief self-report questionnaire consisting of 14 items that has demonstrated validity both as a screening instrument and for evaluating symptom severity [34,35]. The scale comprises two subscales: anxiety (HADS-A) and depression (HADS-D), each containing seven items scored from 0 to 3. Higher scores indicate greater levels of anxiety or depressive symptoms.

***Questionnaires for adults aged ≥18 years with co-occurring intellectual disability***

***Sleep assessment***

Sleep will be assessed using the DASH-II questionnaire [28]. This instrument consists of 30 items, with response options reaching values of up to 5 points, and is designed to evaluate the presence of specific symptoms as well as the individual’s ability to perform certain activities over the previous week [36]. The questionnaire allows the identification of behavioral and emotional symptoms that may affect daily functioning, including those potentially related to sleep disturbances. To assess comorbid conditions with a potential severe impact on sleep, the anxiety and depression subscales of the DASH-II are also administered, providing additional information on emotional symptoms that may influence sleep patterns in individuals with intellectual disability.

### 2.5. Statistical Analysis

In this study, quantitative variables are summarized using means and standard deviations, while qualitative variables are presented as percentages. Descriptive statistics are used to provide an initial overview of the characteristics of the study population based on the common questionnaire (sex, age, educational level, and diagnosis).

The primary outcome is the prevalence and severity of sleep disturbances, assessed using age- and ability-appropriate instruments: Children*’*s Sleep Habits Questionnaire/CSHQ–Autism, Pittsburgh Sleep Quality Index, Sleep Disturbance Scale for Children, and Diagnostic Assessment for the Severely Handicapped–II [24,25,26,27,28]. Sleep outcomes will be analysed both as continuous scores and, where validated cut-offs exist, as categorical variables (presence/absence of clinically significant sleep disturbance).

Secondary outcomes include (i) anxiety and depressive symptoms assessed using age-appropriate tools (Child Behavior Checklist, Hospital Anxiety and Depression Scale, and relevant DASH-II domains) [28,31,34,35], and (ii) their association with sleep disturbances. Additional contextual variables from the common questionnaire (e.g., treatment status, educational level) will also be explored.

Analyses will be conducted within strata defined by age group and intellectual disability status, consistent with the questionnaire structure. Direct comparisons will only be performed between groups assessed with the same instrument and the same type of respondent (self-report vs. proxy-report). No direct comparisons will be made between self-reported and caregiver-reported outcomes due to known differences in reporting patterns. Bivariate analyses will first be performed to explore associations between sleep outcomes and independent variables (e.g., age, sex, anxiety/depression scores, and contextual factors), using χ^2^ tests, t-tests, or non-parametric equivalents as appropriate. Subsequently, multivariable regression models will be constructed within each stratum. All multivariable models will adjust for potential confounders identified a priori (age, sex, and relevant clinical variables from the common questionnaire). The type of respondent (self vs. caregiver) will be included as a covariate or used for stratification, depending on the analysis.

Data completeness will be assessed prior to analysis. Questionnaires with substantial missing or inconsistent responses will be excluded according to predefined criteria. For partially missing data, if the proportion is low (<5%), complete-case analysis will be performed; otherwise, multiple imputation methods will be considered under the assumption of missing at random. Sensitivity analyses will be conducted to evaluate the impact of missing data on the results. Pre-specified subgroup analyses will be conducted by age group and intellectual disability status. To address potential bias arising from mixed informants, sensitivity analyses will be performed by restricting analyses to (i) self-reported data only and (ii) proxy-reported data only. Additionally, where sample size within strata is limited, categories may be combined to preserve statistical power and stability of estimates.

All inferential statistical analyses will be conducted using IBM SPSS Statistics (version 22). Statistical significance will be set at a two-tailed *p* value < 0.05. When multiple comparisons are performed, Bonferroni correction will be applied to reduce the risk of type I error and prevent overestimation of statistical significance.

## 3. Discussion

Sleep disturbances are widely recognized as a relevant clinical concern in individuals with autism spectrum disorder; however, large-scale data in the Spanish context remain scarce. This study is designed to address this gap through a cross-sectional design with a nationwide dissemination strategy, integrating age- and cognitive ability-adapted instruments to capture sleep patterns across the lifespan.

Despite the robust international evidence documenting the high prevalence of sleep disturbances in individuals with ASD, a significant knowledge gap remains within the Spanish context. At present, no large-scale studies have systematically examined the prevalence and characteristics of sleep problems among individuals with autism in Spain. Consequently, little is known about how sleep disturbances may vary according to factors such as age, the presence of intellectual disability, associated comorbidities, or environmental and contextual conditions. The absence of large-scale Spanish data represents an important limitation for both research and clinical practice. Without reliable epidemiological information, it becomes more difficult to design targeted clinical, educational, and social interventions that adequately address the needs of this population. Moreover, the lack of large-scale evidence from Spain may contribute to the continued under-recognition of sleep disturbances as a relevant health issue affecting individuals with ASD and their families [14].

A key strength of this protocol lies in its inclusive design, which allows the participation of individuals with varying levels of functioning, including those with intellectual disability. These instruments include the CSHQ–Autism, SDSC, PSQI, DASH-II, and affective symptom scales such as the CBCL and HADS [25,26,28,31,33]. The use of these validated measures allows for the systematic evaluation of different dimensions of sleep, including sleep quality, sleep disturbances, and the potential influence of emotional symptoms on sleep patterns.

To our knowledge, this study represents one of the first research initiatives in the Spanish context specifically designed to examine sleep disturbances in individuals with ASD using a nationwide dissemination strategy and age- and ability-adapted assessment tools. By adopting a nationwide dissemination strategy, the study aims to provide a broad and geographically diverse overview of sleep patterns and sleep-related difficulties within the autistic population in Spain. In addition, the integration of demographic, clinical, contextual, and affective variables within the same analytical framework allows for a more comprehensive understanding of the multiple factors that may influence sleep in individuals with ASD. Considering these different dimensions simultaneously may contribute to the identification of specific vulnerability profiles associated with sleep disturbances, thereby facilitating a more nuanced interpretation of the complex relationships between sleep, behavioural characteristics, and emotional symptoms in this population.

Several limitations should be considered. First, recruitment through autism associations and online dissemination may introduce selection bias, potentially favoring individuals with greater access to support networks and digital resources. Second, the use of self- and proxy-reported data may introduce measurement variability, although this will be addressed analytically through stratification and sensitivity analyses. Third, ASD diagnosis will not be independently verified through clinical documentation. Although mandatory diagnostic questions and expert clinical review will be used to improve data quality and detect implausible or inconsistent information, this approach is weaker than direct confirmation using clinical records. Fourth, the cross-sectional design precludes causal inference and limits the ability to assess temporal relationships between sleep disturbances and associated factors.

Furthermore, the information obtained through this research may help support the development of more tailored and needs-based care strategies aimed at addressing sleep problems in individuals with autism. A better understanding of sleep disturbances and their associated factors may also contribute to improving screening and assessment practices in both clinical and community settings.

The findings derived from this study are expected to contribute to addressing a critical knowledge gap regarding sleep disturbances in individuals with ASD in Spain. By generating data from a survey with nationwide dissemination, this research may provide an empirical basis for future investigations exploring sleep trajectories across the lifespan. In addition, the results may serve as a valuable reference for the development of public health initiatives and educational policies aimed at improving sleep health within the autistic population. Ultimately, this evidence may support the implementation of multidisciplinary interventions that recognize sleep as a key determinant of well-being, daily functioning, and quality of life in individuals with autism spectrum disorder.

## 4. Conclusions

This survey protocol with nationwide dissemination provides a structured and inclusive framework for assessing sleep disturbances in individuals with autism spectrum disorder across the lifespan in Spain. By integrating validated, age-appropriate instruments and considering both clinical and contextual factors, this study is well positioned to generate relevant epidemiological data in an underexplored setting. The data generated by this study may support future research, inform public health strategies, and guide the development of targeted interventions aimed at improving sleep and overall well-being in this population.

## Data Availability

No new data were created or analyzed in this study.

## Abbreviations

The following abbreviations are used in this manuscript:

ASD	Autism Spectrum Disorder
CBCL	Child Behavior Checklist
CSHQ	Children’s Sleep Habits Questionnaire
DASH-II	Diagnostic Assessment for the Severely Handicapped–II
HADS	Hospital Anxiety and Depression Scale
HADS-A	Hospital Anxiety and Depression Scale–Anxiety
HADS-D	Hospital Anxiety and Depression Scale–Depression
PSQI	Pittsburgh Sleep Quality Index
SDSC	Sleep Disturbance Scale for Children
